# Opening toward life: Experiences of basic body awareness therapy in persons with major depression

**DOI:** 10.3402/qhw.v10.27069

**Published:** 2015-05-07

**Authors:** Louise Danielsson, Susanne Rosberg

**Affiliations:** 1 Department of Health and Rehabilitation, Institute of Neuroscience and Physiology, University of Gothenburg, Gothenburg, Sweden; 2 University of Gothenburg Centre for Person-Centred Care (GPCC), Gothenburg, Sweden; 3 Närhälsan Gibraltar Rehabilitation Centre, Gothenburg, Sweden

**Keywords:** Embodiment, lived body, physical therapy, person-centred care, movement-based therapies

## Abstract

Although there is a vast amount of research on different strategies to alleviate depression, knowledge of movement-based treatments focusing on body awareness is sparse. This study explores the experiences of basic body awareness therapy (BBAT) in 15 persons diagnosed with major depression who participated in the treatment in a randomized clinical trial. Hermeneutic phenomenological methodology inspired the approach to interviews and data analysis. The participants’ experiences were essentially grasped as a process of enhanced existential openness, *opening toward life*, exceeding the tangible corporeal dimension to also involve emotional, temporal, and relational aspects of life. Five constituents of this meaning were described: *vitality springing forth*, *grounding oneself*, *recognizing patterns in one's body*, *being acknowledged and allowed to be oneself*, and *grasping the vagueness*. The process of enhanced perceptual openness challenges the numbness experienced in depression, which can provide hope for change, but it is connected to hard work and can be emotionally difficult to bear. Inspired by a phenomenological framework, the results of this study illuminate novel clinical and theoretical insight into the meaning of BBAT as an adjunctive approach in the treatment of depression.

Depression is a global threat to public health that is steadily increasing in its negative impact and is currently one of the leading causes of functional disability worldwide (Whiteford et al., 2013). Lifetime prevalence is approximately 20% in the Western world, with generally higher estimates in high-income countries (Kessler & Bromet, 2013). Although antidepressant medication and psychotherapeutic interventions are effective treatments, about one-third of people with depression will have an insufficient recovery, encouraging research on adjuvant interventions.

The concept of the life-world refers to the human world of immediately and concretely lived experiences, preceding theoretical explanations (Husserl, 1970/2010). Fundamentally, the life-world is characterized by meaning. According to Merleau-Ponty (1962), the way we can relate to and access this world is through our lived bodies. The lived body is simultaneously both a perceiving subject, and a perceived object, always directed to the world but also already inhabiting it, immersed into it. According to Heidegger's (1953/2010) concept of *Dasein* as a way to understand human existence, humans are thrown into the life-world, “being-in-the-world,” and involve themselves in it by attuning to it. The world and our situatedness in it can only matter to us because we have the capacity to be open to letting things appear and reveal themselves to us, and this openness is the *Dasein*. Drawing on Heidegger, this perceptive and responsive openness to what is encountered is impaired in depression (Aho, 2013; Boss, 1983; Fuchs & Schlimme, 2009; Svenaeus, 2007). This impairment means a narrower range of moods and a more limited way for us to attune to the world. The depressed person feels “corporealized,” encapsulated in his or her body as an object, which limits the potential to engage in life's events (Fuchs, 2005). The whole “bodying forth” (Boss, 1983) of specific human ways of being is restrained. Instead, nothing stands out as significant any longer, as in Heidegger's (1953/2010) analysis of profound “boredom”: “The often persistent, smooth and pallid lack of mood, which must not be confused with a bad mood, is far from being nothing. Rather, in this *Dasein* becomes tired of itself. The being of the there has, in such a bad mood, become manifest as a burden” (p. 131). Related to the life-world perspective, the notion of *Dasein* as the perceptive openness that creates an embodied attunement to and engagement with the world was a theoretical inspiration to the present study.

In our previous work related to the embodiment of depression, we found that the participants’ experiences were essentially captured as an *ambiguous striving against fading*, struggling to resist the urge to withdraw, but simultaneously needing to pause from life (Danielsson & Rosberg, 2014a). The body felt confined, estranged, and heavy, but momentarily a means to sensing life and seeking belongingness. Extending these results, we were interested in the body as the mediator of engagement with the life-world, which directed us toward a treatment perspective.

Movement-based embodied contemplative practices (Schmalzl, Crane-Godreau, & Payne, 2014) have recently received increased attention in the treatment of depression, for example mindfulness meditation (Teasdale et al., 2000), yoga (Balasubramaniam, Telles, & Doraiswamy, 2013; Cramer, Lauche, Langhorst, & Dobos, 2013), tai chi (Lavretsky et al., 2011; Tsang et al., 2013), and dance and movement therapy (Koch, Kunz, Lykou, & Cruz, 2014). The suggested working mechanisms are biological and neurological mechanisms that are shared by other types of physical activity, but recent theories also highlight neurophenomenology and enactive dimensions (Schmalzl et al., 2014).

The construct of body awareness refers to a particular kind of mindful, non-judgmental awareness and a sense of self, grounded in physical sensations in the present moment (Mehling et al., 2012). Scandinavian physical therapists use a treatment method called BBAT, which aims to enhance awareness of body and self through exploration of basic movement principles such as functional balance, free breathing, mental awareness, and embodied presence (Lundvik Gyllensten, 2001; Skjaerven, Kristoffersen, & Gard, 2010). BBAT involves both activating and experiencing one's body through movement, acknowledging that these experiences touch different dimensions of existence: physical (like the organic matter of bones and muscles), physiological (processes such as blood circulation), psychological (attention, emotions), and existential (the sense of “I am”) (Hedlund, 2014). The theories of BBAT have been mainly inspired by the works of Swedish physical therapist Roxendal and French movement educator Dropsy; they are influenced both by Eastern movement traditions such as tai chi chuan and Zen meditation and by Western traditions such as dance and movement pedagogy (Hedlund, 2014). BBAT has been found to be beneficial for various disorders, for example long-term musculoskeletal pain (Malmgren-Olsson, Armelius, & Armelius, 2001), fibromyalgia (Gustafsson, Ekholm, & Broman, 2002), irritable bowel syndrome (Eriksson, Möller, Söderberg, Eriksson, & Kurlberg, 2007), schizophrenia (Hedlund & Gyllensten, 2010), and eating disorders (Catalan-Matamoros, Helvik-Skjaerven, Labajos-Manzanares, Martínez-De-Salazar-Arboleas, & Sánchez-Guerrero, 2011). In psychiatric out-patients, a period of BBAT improved sleep, self-efficacy, and physical coping resources (Gyllensten, Hansson, & Ekdahl, 2003a). Patients’ experiences of BBAT related to increased balance, awareness, handling of body signals, and movement control (Gyllensten, Hansson, & Ekdahl, 2003b). Their self-confidence grew as they developed sensitivity to body cues and mastering their bodies in movement. The authors concluded that the patients’ experiences of the relationship with the physical therapist seemed to affect their view of the meaningfulness of BBAT. The patient's ability to open to and trust the physical therapist was suggested to be important for a positive outcome (Gyllensten et al., 2003b). In a similar way, schizophrenic patients described enhanced awareness and self-esteem following BBAT (Hedlund & Gyllensten, 2010). They also noted that BBAT helped with affect regulation, social abilities, and thinking more clearly. Another study on patients with moderate psychiatric illness and pain found that BBAT provided a situation to create new meanings for one's lived body, as the patients experienced their bodies in a new way, feeling more whole and “at home” with themselves (Johnsen & Råheim, 2010). This meant an expanded self-knowledge and changed ways to relate, but it was connected to thresholds because the changes took time.

Studies on BBAT in mental health often involve mixed samples, indicating a need to address what it is like to take part in BBAT while enduring a specific illness. Moreover, a life-world-based research perspective for studying BBAT is rare. A recent randomized controlled trial evaluating BBAT for major depression indicated that more research is needed both to conclude effects and to deepen the understanding of how BBAT is experienced during depression (Danielsson, Papoulias, Petersson, Carlsson, & Waern, 2014b). Thus, for the present study, we were interested in lived but unpronounced aspects of BBAT during depression, how BBAT is lived through and given meaning to by persons who are depressed. To gain insight into this experience, we phrased the following research question: What meanings do persons with major depression give to their experiences of BBAT? The purpose of this study was to explore the phenomenon of BBAT as experienced by persons with major depression.

## Method

Hermeneutic phenomenology is concerned with reflection, in an open and wondering way, on the basic structures of the lived experience of human existence to achieve access to the world as we experience it pre-reflectively (Van Manen, 2014). This study commits to the life-world of persons diagnosed with depression, searching for meaning in their immediate and intuitive experience of BBAT as a wondering approach to *what shows itself* in this experience and *how it shows itself*. By “borrowing” their experiences of BBAT and allowing ourselves to be absorbed by them, we can enrich our understanding of what the experience means. This endeavour involves the phenomenological reduction, or what is referred to by others as *bracketing* or *epoché* (Norlyk & Harder, 2010). These expressions signify withholding existential claims of the phenomenon by suspending previous ideas and prejudices, so that the phenomenon can be approached with an open mind. However, to grasp embedded meaning beyond pure description, the researcher's stance and active collaboration with the participants inevitably involve an interpretative dimension (Dahlberg, Dahlberg, & Nyström, 2008; Van Manen, 2014). Here, we concur with Heidegger's (1953/2010) and Gadamer's (2004) views of interpretation as always founded upon our preconceptions; without these we can never apprehend anything. Thus, our reflexivity as researchers, with our own experiences, knowledge, and beliefs, and the interrelation between researcher and participants were dynamically intertwined with the generation and analysis of data.

In phenomenological research, the vocative and linguistic dimension is central to the analysis, especially to the writing (Van Manen, 2014). We worked to embrace, on the one hand, a systematic exploration of the meaning structures and, on the other hand, an attempt to evoke resonance in the reader. This means that we sought language that would capture the experiences in a way that would create a certain sense in the reader. The experiences described should feel plausible, recognizable even though the reader has not experienced them personally. Thus, the language needs to be rich and creative, to make something understandable that, in fact, lies beyond spoken and written language—ideally, as Merleau-Ponty (1973) says, “a language that sings the world.”

### Participants

In phenomenological research, sampling is not related to statistics or to saturation in the sense that no additional views appear (Van Manen, 2014). Rather, sampling means gathering enough examples of experientially rich descriptions to be able to help us connect to life as it is lived (Van Manen, 2014). Here, we included 15 participants diagnosed with major depression^1^ according to the criteria of the Diagnostic and Statistical Manual of Mental Disorders, 4th edition (American Psychiatric Association, 2000), who had taken part in a BBAT intervention in a randomized controlled trial presented elsewhere (Danielsson et al., 2014b). The severity of depression was the primary outcome, measured using the Montgomery Åsberg Depression Rating Scale (Montgomery & Åsberg, 1979). In the trial, 62 adults with major depression were randomized to participate for 10 weeks in one of the three following interventions, in addition to antidepressants: 1) exercise, 2) BBAT, or 3) advice on physical activity in daily life. Experienced physiotherapists guided the interventions. Effects on psychological, functional, and physiological parameters were compared across groups. In the BBAT group, participants began treatment with two individual sessions and then trained for 8 weeks, twice weekly, in small groups of five to eight participants. For this qualitative study, we attempted to recruit participants who had attended at least five sessions altogether (individual and group sessions), because we assumed that, with fewer sessions, it would be difficult for participants to express experiences of treatment in a way that would provide rich descriptions. Among the 20 participants randomized to BBAT in the intervention study, 12 were initially invited to take part in the present study. We eventually included a thirteenth participant who was originally in the control group, but subsequent to follow-up received a similar program of BBAT. The reason for this inclusion was that we wanted another example of experiences to move our understanding further, particularly the experiences of a young person because the sample contained few young people. During the analysis, we considered the idea that a negative treatment experience would give important nuances to the results, and we therefore contacted two participants who had dropped out during the trial; these participants provided us with written recounts of their experiences. However, one of these participants had attended fewer than the stated minimum number of sessions (three sessions in total). The participants represented a variation of background characteristics (sex, age, occupational and marital status, baseline depression severity, and number of sessions completed). The demographic and clinical data of the participants are presented in Table I. The structure and main content of the BBAT intervention are presented in Table II.

**Table I:** Background and clinical data for the participants included in a study exploring experiences of basic body awareness therapy in persons with major depression

		Number of participants Total sample *n=*15
Age	Median age=47, range 19–64	
Sex	Women	10
	Men	5
Occupational status	Full-time work	3
	Part-time work	5
	Student	3
	Full sick-leave/pension	3
	Unemployed	1
Depression severity before intervention	Median MADRS=22 points, range 15–27	
	<20 MADRS points	5
	≥20 MADRS points	10
Number of completed sessions (max 18)	Median=13, range 3–18	
	Fewer than 5 sessions	1
	5–10 sessions	5
	11–18 sessions	9
Time from end of intervention to the interview	Median time (weeks)=2, range 1–14	
	<2 weeks	7
	2 weeks–1 month	6
	1–3 months	2

MADRS, Montgomery Åsberg Depression Rating Scale (Montgormery & Åsberg, 1979), a 10-item assessment with a maximum score of 60 points. Generally, scores below 20 indicate mild depression, 20–35 points signifies moderate depression and >35 points represents severe depression.

**Table II:** Content and structure of the basic body awareness therapy intervention that was explored in a study of participants’ lived experiences of the treatment

Structural frame of each session			Examples of exercises	Main purpose
Preparation phase (voluntary)	15 min before session	Room open, mats on the floor, pillows available, calm background music	Resting in sitting or lying down position	Calming down, preparing for session
Phase 1	About 20 min	Supine movements on the floor, on mats	Body scanning, exploring contact with the ground and breathing pattern, stretching and releasing movements	Grounding, relaxation, and connecting to one's body
Phase 2	About 20 min	Standing and walking exercises	Balancing in and exploring a functional posture and wholeness, for example: slowly moving up and down along one's midline, flexing in the knees and hips, letting the arms float up when rising, and softly sinking down when lowering one's body, integrating the whole movement with breathing	Postural stability, flow and rhythm of movements, force, coordination
Phase 3	About 10 min	Seated meditation	Aligning and anchoring oneself in a seated position on a meditation cushion or stool, 5 min silent focus on features of the body, such as the breathing	Postural stability, free breathing, mental awareness
Phase 4	5–10 min	Verbal reflection	Taking turns to share something about today's experiences, answering the question: What did you notice during training today?	Sharing and verbalizing body experiences

### Interviews

Semistructured interviews were conducted at the health centre at which the intervention had taken place, as we assumed that recalling experiences would be easier at the locale of the treatment. An effort was made to use the actual training room for the interviews, although this was not always possible due to other regular activities at the centre. Moreover, it was hoped that using a room that was familiar to the participants would facilitate an atmosphere of trust. The interviews were held in the style of a conversation to encourage the participant to talk freely about her or his experiences. Emphasis was placed on creating a collaborative relationship with the interviewer (LD) acting as an interested guide, using follow-up questions to help the participant elaborate and reflect on the experiences described. Two topic areas were employed: 1) experiences of BBAT during depression and 2) narrative about a situation or situations from BBAT that the participant particularly remembered. After the first four interviews, to enhance the immediate experiences, the interviewer began offering to lead the participants in some BBAT movements. Four participants chose to take part in this. This experience deepened the subsequent verbal reflection for one participant, although for the other three, we found no clear advantage to this strategy in terms of facilitating richer descriptions. The interviews were audio-recorded with a digital device and lasted 35–75 min. The interviewer also took working notes to remember key expressions in the participants’ statements and to note non-verbal elements of the interview such as body language, emotional expressions, tone of voice, and atmosphere in the room. Shortly after each interview, the interviewer transcribed the recording verbatim.

### Data analysis

Initially, both authors independently read the transcripts to get a sense of the whole material. The first impression of meaning that came forth in these brief readings was noted in the reflexive journal, together with working notes and thoughts emerging from the interviews. Next, each transcript was analysed closely, on its own terms, which meant thorough readings and systematic structuring of the textual data using tables (see Table III, for example), extracting statements, condensing the meanings involved, and reflecting on the structure of embedded meanings. Reflective discussions between the two authors enhanced this part of the analysis. The discussions were extended by talks with the physiotherapy leader of the BBAT group, who had observed, moved, and reflected with the participants, and by connecting to LD's experiences from leading BBAT groups with other depressed patients. Parallel to the discussions, readings of comprehensive literature inspired a deeper interpretation of the data. However, in accordance with our phenomenological approach, we wanted to put theoretical models “on hold” so that meaning would arise from descriptive, empirical data. The analysis continued through an iterative process of moving back and forth between the separated meaning units and their meaning structure and between each individual interview and the material as a whole. With this analytic movement, we sought a thematic structure and presentation that would capture and lie close to the participants’ descriptions yet reveal meaning beyond these descriptions, enabling us to understand them in a novel way, with the theoretical framework as a tool in the interpretation. Here, the analysis resulted in an essential theme with five constituents that illuminate the meaning of the participants’ experiences of BBAT.

**Table III:** Example of structural analysis moving from meaning units to thematization in a study exploring lived experiences of basic body awareness therapy in persons with depression

Meaning unit	Condensation		Constituent	Theme
I walk in a different way, when I feel bad it's so hard to go from one place to another, stiff like a tin soldier, but now I can sort of spring off the ground … yeah it's a difference all right.	Different walk, from stiff to springy	Springiness	Vitality springing forth	OPENING TOWARD LIFE
I noticed that I started to yawn uncontrollably the last sessions with the group! [laughs] Really, I couldn't hold the yawnings back and it felt great just to release them, loud and big yawning, 'cause it felt so good for my body. But before, I held all such things back.	Yawning uncontrollably, releasing instead of holding back	Releasing body functions	Vitality springing forth	
I discovered, to my surprise, that I've felt happy—quite often lately. Something that I haven't felt in a long time. Some kind of … satisfaction, a happy feeling.	Surprisingly happy	Happiness emerging	Vitality springing forth	
You became aware of your body. It struck me in various situations when I could feel that I was suddenly tensing up, that I had never realized before.	Becoming aware of when I'm suddenly tensing up	New awareness of patterns	Recognizing patterns in one's body	
Yeah, that's why I came to think about it … We started doing it and then I said, well I remember I used to do like this …	Came to think about what used to make me feel safe	Body memories from	Body memories from movements	
But it was unconsciously, I didn't think of it before or analyze it, but I really felt safe, that's why I did it ….		movements		
Now I understand more and more, the connection between how I feel and … well, the contact I have with myself and how my stomach reacts … I realize something or that I learn to interpret my body and listen more to myself, sort of.	Connection between self and stomach, learning to interpret my body	Insights about one's bodily self	Recognizing patterns in one's body	

### Handling of own subjectivity in the process

The researcher's subjectivity is a means to understand the participants’ experiences, both regarding what can be considered relevant in the data collection and as a tool in the interpretation process. On the other hand, that subjectivity needs to be reflected on in order to be able to “bridle” it (Dahlberg et al., 2008), in the sense of staying open to letting the participants’ descriptions lead the way to disclosing meaning. The pre-understanding at hand was that LD is a physical therapist specialized in treating patients with mental health problems in psychiatric and primary care settings; she is educated and trained in BBAT. She has previously done research on the embodiment of depression from a phenomenological perspective. SR is a physical therapist experienced in psychiatric physiotherapy and in body awareness therapy, currently involved in education and research, and with a theoretical foundation in hermeneutic phenomenology and dance and movement therapy. As a tool in the reflective process of keeping openness to novelty and the participants’ voices, we outlined our conscious preconceptions in relation to the research question in a reflexive journal. During the study, we went back to reflect on these assumptions and their relation to the emerging results. Table IV gives an example of the work with our pre-understanding during the analysis process. To further promote phenomenological openness, we engaged in an attentive and empathic presence in the interaction with the participants, both regarding the collaborative style of interviewing and in terms of embodied, non-verbal communication attempting to reach an empathic understanding of the participants’ experiences.

**Table IV:** Example of the analytical process of challenging and transcending the researchers’ pre-understanding in relation to the constituent *vitality springing forth*

Pre-conceptions outlined	Experiences in the data	Reflection	Reformulated understanding
*Experience-based*			
In movement, something seems to happen with the person, like a clarity or awakening, seen in the eyes and body expression.	The last sessions I found myself yawning uncontrollably. I just couldn't help myself. And it felt great!	The movements “stir” something up?	BBAT seems to “poke” at the standstill experience of depression, by sparking
*Theoretical*			both pleasurable and less
The body in depression feels numb.	It was like pinching holes in my armour, and out came a playfulness that I hadn't felt in a long time. Where did this anger come from?	The numbness is like a shell, which can be altered? An often, but not always welcome “emancipation”? Pleasure AND discomfort	pleasurable signs of vitality springing forth
Energy can be released by grounding of the body and freeing of the breathing.	You calm down and you land … my body gets rolling and swings by itself; it sort of goes “wrrm,” back and forth without having to make an effort.	Energy follows the “landing” process but is also much connected to rhythm	BBAT's grounding but also *rhythmic* movements open up for vitality emerging

BBAT, basic body awareness therapy.

### Ethics considerations

When interviewing persons who are particularly vulnerable, as in this case due to mental illness, careful consideration is important to avoid making their health worse. First, none of the participants in this study had an imminent risk of suicide or self-harm, according to the diagnostic interview prior to the intervention. Second, all participants had ongoing contact with health professionals. Third, we believed that the interview style, encouraging participants to narrate in their own way, would allow each participant to choose whether or not to talk about sensitive topics. The study followed the ethical principles of the World Medical Association Declaration of Helsinki (2013) and was approved by the regional ethics review board on March 5, 2012, registration number 027-12.

## Results

The analysis resulted in the essential theme—*opening toward life*—illuminating the meaning of the participants’ experiences of BBAT. This theme is presented below, with five constituents that express different facets of its meaning: *vitality springing forth*, *grounding oneself*, *being acknowledged and allowed to be oneself*, *recognizing patterns in one's body*, and *grasping the vagueness*.

### Opening toward life

The meaning of the participants’ experiences is understood as a process toward enhanced perceptual openness to different aspects of life. The participants expressed feelings of being cut off from the body, self, and world; their descriptions of BBAT relate to a rediscovery of the body as a means to connect to oneself and to life. According to the participants, a key to opening is to direct the focus toward specific features of the concrete body, which shifts the attention and breaks through ruminating thoughts. Their immediate sensations during training made them perceive the body as changeable and dynamic, and this experience sparked something within—*moved* them—in an opening direction. In a concrete way, the experience touched something within them and meant a reaching toward others, being emotionally able to take in more of the outer world. The experience of opening is reflected in such metaphors as “like pinching holes in my armor” and “coming out of my bubble.”

In both pleasurable and disturbing ways, the movements awakened and shook things up, challenging the participants’ experience of numbness and dissipation. Sceptical at the beginning of treatment, some participants came to sessions out of a sense of duty, but many found themselves gradually entering a particular present and relaxed “mode” of accessibility and attunement when entering the room before a session.

However, the training not only opened to enhanced access in a positive manner, but also to emotional distress, provoking anxiety and stress over the silent, slow movements. Some participants were struck and afflicted by the heaviness that sometimes invaded the room during group sessions. These uncomfortable experiences were clearly expressed by the two participants who chose to drop out of the treatment due to discomfort. Others experienced feelings of sadness and distress, but found that they were able to reflect upon these emotions, process them, and consequently obtain new personal insights.

#### Vitality springing forth

This constituent of the theme concerns the experiential opening as increased vitality and invigoration, that is, seizing signs of life through the body. This contrasts with the participants’ experience of a detrimental draining of energy during depression. They recalled instant vital responses to the exercises, such as being able to breathe more freely, spontaneous sighs and yawns, walking more vigorously, or sensing the elasticity of the muscles when stretching and releasing them. Another aspect of vitality was the experience of oscillating flow and rhythm in movements, connecting to elasticity and lightness, the body moving freely, “as by itself”:

Like the movement in standing, swinging the arms, like a rotation. It feels good, it's like my body gets rolling and swings by itself, it sort of goes “wrrm,” back and forth without having to make an effort, it goes by itself.

Mainly, the vitality connected to feeling uplifted or harmonic, but there was also a release of unpleasant emotions, such as anger or sorrow, that could spring forth quite unexpectedly:

Once when we were sitting on those meditation cushions, we were facing the wall and relaxing and sitting upright and all that. And I suddenly felt as if I was going, I almost got angry, mad! If anyone had said something to me, I could have just punched them! That's how it felt (giggles)! As from nowhere it just came. The feeling was there, I just sat through it, but I remember that I was very fascinated, where did this strong anger come from?

#### Grounding oneself

Another aspect of openness concerns the physical and existential experience of letting down one's weight, as a settling ground to start from, an experience connected to rootedness. Paradoxically, although the participants depicted depression as heaviness, they ascribed a positive meaning to the experience of the weight of their bodies during training, in standing, sitting, or supine movements. The participants associated this grounding experience of the weight of the body to a “landing platform” of certainty and stability, rooting oneself in reality and familiarity. Here, the experience of gravity, of being supported by the ground, is related to firmness and reliability, as earthly, immediate evidence of one's existence rather than a restraining heaviness: “Well, I feel lighter but heavier! [laughs] … It's hard to describe in words, but it feels like you are more … whole. A whole. You feel lighter but at the same time more stable.”

Also related to rootedness was the participants’ experience of spatially and rhythmically shaping and coordinating movements, “rather than feeling like a jumping jack.” This sense of managing one's body, trusting that the action will follow the intention, connects to a higher embodied trust. The challenge of exploring unfamiliar movements can be fruitful as the gradual embodied trust relates to feeling at home with oneself. One young man described his metaphorical experience of trust from a relaxation exercise in the following way:

It was like finding a clearing in the woods, entering my calm place, my happy place. It was like coming to a place where you just smile. To go there, I have a mental picture of a long stairway that I descend. Like sinking down to this calmness. I see another figure, maybe that's also me, looking back at me, smiling—like a welcome. And it's easier now to find it again, now when I meditate I can also find it. Sometimes I start in the stairs but I don't get all the way down and sometimes I'm just down there and I don't have to search 'cause I know where it is. It feels a little like when you are about to fall asleep and you feel like falling, but in the nice way, like when you know you're gonna have a good night's sleep.

#### Recognizing patterns in one's body

This constituent concerns the participants’ experiences of opening for a recognition of the close relationship between their physical and psychological distress. They described noticing how they were moving—posture, tension, and breathing—and they linked these patterns to how they felt emotionally. Exploring immediate bodily sensations during sessions made them aware of personal patterns, such as becoming tense in certain stressful situations or realizing that one has a tendency to exert oneself. The insights and “know-how” of the movements are not without effort—it takes time for the penny to drop, as one participant explained. Most participants described “aha moments,” when they suddenly recognized something that made sense to them, such as one woman who shared her insight that she tends to walk briskly and stomp in the street when in fact she feels angry.

The participants also associated their immediate experiences to previous life events, linking the “now” with the “then” of their bodies. For example, one woman recalled with longing sports activities she used to do as a child, enjoying playing on a football team. A young woman shared a reminiscence of her childhood, connected to her experience of a standing, sideways movement:

It was when we were trying out the standing movements, there was something, I think it was when we rocked a little from side to side and then I came to think about what I did instinctively when I felt really bad—I used to sit rocking my whole body like this [rocks back and forth in the chair with arms crossed]. But that was unconscious, I didn't think about it or analyze it until now, but it, that was really comforting, that's why I did it … and the rocking, I don't know … as a child I used to love being in the swing. So I think that when I move like that, it calms me down, I can do it now [rocks again], just a little movement makes a difference.

#### Being acknowledged and allowed to be oneself

This constituent involves experiences of a relational openness associated with a trusting and accepting atmosphere, in the room, in the group, and in the verbal and non-verbal dialogue with the physical therapist. The participants felt that they were allowed to be themselves, with their difficulties, peculiarities, and all, and that they were seen for who they are. Initially feeling reluctant to interact—one woman preferred the full attention of the physical therapist while another said that she was happy to “mesh” with the group to feel less exposed—the participants gradually opened up for coherence and sharing of experiences:

Since we met twice a week, we got acquainted with each other in a way. And that became a trustfulness in the group. It was good for me, luckily it wasn't a big group like an aerobic class with thirty people or more bouncing around in the room. I doubt that I would have felt comfortable with that. But this small group … calm and sensible with no demands for achievements.

Being acknowledged also influenced the participants’ relations with the physical therapist. They claimed that the professional support was necessary for them to attend regularly and to maintain their motivation. Some recalled bad experiences from other encounters in health care, where they felt diminished or ignored, physical therapists with mechanistic views of their bodily distress or group leaders who were too vague. They described feeling, here, that they were seen in a warm and tolerant way, often without words, for example when the physical therapist saw the need for an extra pillow or gave a gentle pat on the back if a participant was feeling particularly low. The therapist's guidance, mainly moving together with the group, gave direction and purpose:

I appreciated the professionalism in the leading of the group. It was never gooey, that's my word, 'cause I'm allergic to gooey. No, there was warmth and concern, but there was also a “here and now.” Now we do this and now we do that, but still in a receptive way. And that was great for me.

#### Grasping the vagueness

This constituent concerns the participants’ experiences of grasping the experience of vagueness, by actually “doing something rather than just floating along indifferently.” It is not just about being aware of one's body but about trying something for oneself, by the act of moving, that becomes a foothold in the vagueness and incapacitation of depression. The sense of grasping the vagueness clarifies and discerns, as a defining of one's participation in one's own life. The participants emphasized that it was hard and sometimes painful work and that they had to surpass an enormous depth of resistance, “like passing through the Mariana trench,”^2^ as one woman said. In this sense, ability and vulnerability were closely linked for the participants:

Something is bound to happen, I feel like I try so hard. And when I doubt myself, at home, I say to myself that, well it might sound ridiculous, but well, at least I'm trying this now, I'm *doing* something.

Some participants started to use parts of the movement sessions, modified in their own personal ways, as coping strategies in daily life. The experience of grasping can transcend the treatment room also in the sense of allowing agency in a broader sense:

The last two weeks of the course I felt more determined, in general. I thought that now when this is ending, it's up to me to find other positive activities, maybe it's time for some change. Switch some things around in my life, like, how do I spend my days? Should I really do that, or should I try and finish some things and, well, try something new. And that's the work I have to do now, I feel that this process has started.

## Discussion

The main results of this study highlight the multidimensional process of perceptual opening toward life as the essential meaning of BBAT as experienced during depression. This opening motility goes beyond the manifest physical expression of movement to also involve emotional and existential dimensions of one's being in and with the world. This process can be interpreted in light of Heidegger's (1953/2010) notion of the *Dasein* as the embodied attunement to the world—what we open and close ourselves to—that in turn constitutes the possibilities of what can shine through and create meaning. Because in depression this openness is, to quote Heidegger, “tired of itself,” the experience of bleakness and insignificance permeates a person's attunement to the world during depression. With regard to our findings and the meaning of BBAT as opening toward life, we suggest that participation in BBAT creates a possibility to widen, or at least knock on the door of, this narrowed attunement.

Similar to previous qualitative studies of BBAT, we found that the exercises’ concentrative focus on noticing details of one's body was useful for improving a sense of presence (Johnsen & Råheim, 2010). Moreover, in line with previous work, our results revealed the significance of mastering one's body (Gyllensten et al., 2003b; Hedlund & Gyllensten, 2010), but connected to the “doing” of BBAT, as a means to agency rather than to self-control. Previous studies found that BBAT evoked distress in terms of unpleasant but necessary feelings (Hedlund & Gyllensten, 2010) or feelings that were demanding and time-consuming to process (Johnsen & Råheim, 2010). In the interpretation of our data, we acknowledged that for some participants, especially the two participants who had quit the treatment, there was an aspect of profound, threatening distress that was difficult to articulate, almost like an emotional abyss or “the Mariana trench,” as one woman said. Possibly, this relates to the hollow and estranged experience of depression (Danielsson & Rosberg, 2014a). This aspect needs to be further studied to aid in understanding clinical implications.

### Temporal “elasticity”

Our findings relating to recognition suggest a temporal dimension in BBAT, promoting an extended temporality through the body. This phenomenon is exemplified by one of the quotes illuminating the constituent *recognizing patterns in one's body*, with a participant immediately recalling childhood memories while performing a particular movement. Our interpretation of this phenomenon is that the awareness of the movement, based on sensory and motor cues and discovered in co-creation with someone else (i.e., the physiotherapist or other participants), seems to transcend the here-and-now of embodied experience to traverse the phenomenological, lived time, connecting instantly to other life-events. Staying in the movement with conscious, yet relaxed attention seems important, giving this temporality room and, in a way, surrendering to it to allow it to unfold. Also, moving together, with guidance, seems to enhance this lingering awareness, which requires and simultaneously develops a basic sense of embodied trust.

The temporal domain of depression has recently been given increased attention by studies pointing to the stagnated, frozen quality of lived time as a key feature (Aho, 2013; Fuchs, 2013), fettering the possibility of reaching out with the *extended body* (Froese & Fuchs, 2012) and inhibiting the ability to relive and reconnect experiences across one's past, present, and toward the future. For this reason, the opening for temporal “elasticity” that the participants in our study experienced (such as remembering past experiences and making sense of these in the present) is particularly relevant and provides an area for future research. The temporal elasticity in the experiences of BBAT, expressed through immediate embodied recognition, recalling, and verbalization of life-events, could be regarded as an aspect of body memory (Koch, Caldwell, & Fuchs, 2013). Our results can also be related to the recent studies of Norwegian Psychomotor Physiotherapy, which illuminate patients’ process of transformation through new experiences in movement and in sensation, with the new experiences feeding their narrative imagination and reshaping past plots, embodied identity, and future prospects (Sviland, Martinsen, & Råheim, 2014; Sviland, Råheim, & Martinsen, 2012).

### Challenging and enhancing *“homelikeness”*

Our findings reveal bodily sense of rootedness as “feeling at home” with oneself in the world, in an effortless, low-key manner, expressed in the constituent *grounding oneself*. The concept of *unhomelikeness*—the experience of unhomelike being-in-the body and being-in-the world—has been proposed as a way to understand the experience of illness (Gadamer, 2011; Öhlén, Ekman, Zingmark, Bolmsjö, & Benzein, 2014; Svenaeus, 2011). When struck by illness, in particular long-term conditions, the person's lived body will change, altering his or her ways to relate to the world. The previous well-known, pre-reflective experience of one's body, being meaningfully situated to act and relate, is suddenly or gradually characterized by unfamiliarity and estrangement. In philosophical analyses, depression and anxiety have served as core examples of unhomelikeness (Svenaeus, 2011). Interestingly, our findings suggest that BBAT might both challenge and enhance the embodied trust and a homelike feeling of oneself. The notion of homelikeness may be a useful contribution to the future development of the concept of body awareness (Mehling et al., 2012).

### Relating to oneself and to others

Our results convey an essential relational sphere in BBAT, understood as two-dimensional. First, the results propose an intra-subjective dialogue between *I* as sensing subject and *me* as the object to sense, as for example in the participants’ descriptions of noticing concrete details on a physiological level to feel more present and able to take in outer impressions, or in the increased awareness of the self—emerging from observing objective features of one's body and movement (Danielsson, Hansson Scherman, & Rosberg, 2013). Second, the numerous statements about the growing coherence in the group and the meaning of embodied communication with the physical therapist suggest an interrelational dimension, also previously suggested (Gyllensten et al., 2003b). These dialogues between *I* and *me* and between *I-me* and other persons might develop in future research by drawing on the double intentionality of the lived body as object-subject directed to the world (Merleau-Ponty, 1962). However, what we found striking in our data was the meaning related to being seen as a capable person, but with acknowledgment of one's incompleteness and difficulties. According to Ricoeur (1994), being human means that we are always constituted both by potentiality and vulnerability and this dialectic is what makes us capable and genuinely human. This view, central in a person-centred approach to rehabilitation (Ekman et al., 2011), moves the focus from a professional expert trying to solve the individual's problem to a dialogue between able/vulnerable, responsible, and relating persons.

### Strengths and limitations

Throughout the different phases of the study, we were guided by Van Manen's (2014) validation criteria for phenomenological research, which involve the following aspects: that the research question should ask about what a particular experience is like or how it is lived; that the analysis should be conducted on experientially descriptive accounts; that the study should be rooted in primary and scholarly phenomenological literature; and that the study should avoid attempts to legitimate itself using unsuitable validation criteria.

Depth and recognizability in the descriptions are facilitated by richness and expressiveness in the data (Van Manen, 2014). In our previous research analysing narratives of persons enduring depression, we had noted that the depressive state seemed to disable the person's recalling of anecdotes and narrowed the imaginative aspects of their language. Thus, for the present study, the interview focused on the participant's description of a concrete, memorable moment (Van Manen, 2014) from treatment, using follow-up questions to guide the narrative toward sensory dimensions (for example, asking about the ambience in the room, the sense of the floor, the presence of emotions). The possibility of doing BBAT movements together during the interview was thought of as enabling more immediate, “lived through” experiential descriptions. This strategy did not appear to be particularly helpful in facilitating descriptions; rather, a gap remained between verbal accounts and tacitly working with the body. However, the interviewer got insight, from a second-person perspective, into how the participant moved and became immersed in the exercises. This experience provided an embodied dimension for the interpretation, although not part of the narrative data. For future studies, an alternative could be to let the researcher silently participate in and observe during the actual treatment, taking field notes afterwards. We found that the participants’ descriptions in the present study were expressive enough to capture a “sensed,” near understanding (Van Manen, 2014). It is also notable that the depressive state of several participants was alleviated during treatment, possibly enabling a richer language.

To enhance the interpretative movement by involving a dialogue with theory, we turned to the works of Heidegger and Merleau-Ponty, but also to novels and poetry relating to the experience of depression. Moreover, the interpretative process was deepened through reflective talks with the BBAT group leader and LD's parallel clinical practice.

Transparency of the research process was enhanced through a visual presentation (Table III) of the structural analysis, to clarify the link between data and meaning structure and through concretizing the process of reflecting on our preconceptions (Table IV). We also put effort into the linguistic presentation (Van Manen, 2014), maintaining closeness to the participants’ language and their use of metaphors, to convey to the reader what we captured as expressive of the embedded meaning. That being said, the usefulness of the results is always in the eyes, mind, and body of the reader.

The recruitment of participants from a randomized controlled trial comes with challenges, mainly because it limits the sampling. It can be argued that none of the participants had genuinely long and vast experiences of BBAT; they had attended at most 18 sessions over a 10-week period, with a median of 13 sessions. This somewhat conflicts with the phenomenological attitude, which seeks to borrow *examples* of experiences of a phenomenon, in order to understand it as fully as possible. For the same reason, it can also be argued that five attended sessions was a low minimum for inclusion. However, we found that the number of sessions attended by a participant did not correspond to how explorative and dense their narration was. For example, one interview that we perceived to be deeply evocative, concretizing and yet opening our understanding of the phenomenon, was conducted with a participant who had attended only six sessions. On the other hand, it can also be argued that the choice to include only participants who had attended at least five sessions could mean that persons who dropped out early were not included, which might result in an overly positive presentation of BBAT. Hence, we added the written accounts of two participants who had dropped out. Moreover, the results show a complexity of openness, for example by coming into contact with overwhelming emotions of fear and anger (see the constituent *vitality springing forth*), or by painful insight into how one has cast aside one's physical needs (see *recognizing patterns in one's body*).

Another limitation can be pointed out in relation to the research team. We stress the importance that the researchers possess previous, personal knowledge of BBAT, since the verbalization of embodied experiences can be difficult. Our pre-understanding was thus useful for enhancing our *sense* of the participants’ narrations and building a collaborative approach during the interviews. However, another researcher with a different profession might have contributed other perspectives. As a peer validation, preliminary results were reflected on with clinical collaborators and researchers from other fields of health-care sciences.

### Implications

This study is the first to explore experiences of BBAT in a study sample of persons diagnosed with major depression. In this sense, the results provide insight into what BBAT can mean for persons with depression and give some direction to clinicians regarding as to how the treatment may be useful. For physical therapists, the results may inspire reflection on a novel frame of phenomenological interpretation as a step toward further developing the theoretical framework of BBAT, and they may illustrate obstacles and possibilities in the treatment process. On a theoretical level, we believe that the findings might add to the discussion of the construct of body awareness, for example to elaborate on the dimensions of temporality and homelikeness.

## Conclusion

Experiences of BBAT in persons with major depression are essentially grasped as a process of enhanced existential openness, exceeding the tangible corporeal dimension to involve also emotional, temporal, and relational aspects of life. These glances of openness toward life challenge the numbness and incapacitation experienced in depression and may provide hope for change, but they are connected to hard work and can be emotionally distressing. Inspired by a phenomenological framework, the results illuminate novel clinical and theoretical insight into the meaning of BBAT as an adjunctive approach in the treatment of major depression.

## Notes

1 Major depression is the psychiatric term for a clinically relevant depression. Causing significant impairment in occupational and/or social life for at least 2 weeks, one of the two core features, 1) depressed mood and 2) loss of interest, must be present for a diagnosis. Moreover, at least five of nine symptoms regarding mood, interest, appetite, sleep, activity, energy, concentration, feelings of guilt, and suicidal ideations, should be present.

2 The Mariana Trench is the deepest part of the world's oceans, located in the western Pacific Ocean close to the Mariana Islands.
